# An Adolescent Case of Anti-MDA5 Antibody-Positive Juvenile Dermatomyositis With Interstitial Lung Disease Successfully Treated by Multitarget Therapy Avoiding Cyclophosphamide: A Case Report and Literature Review

**DOI:** 10.7759/cureus.62425

**Published:** 2024-06-15

**Authors:** Tadafumi Yokoyama, Natsumi Inoue, Naoto Sakumura, Yuko Tasaki, Taizo Wada

**Affiliations:** 1 Department of Pediatrics, Kanazawa University, Ishikawa, JPN

**Keywords:** multitarget therapy, mycophenolate mofetil, tacrolimus, anti-mda5 antibody, juvenile dermatomyositis

## Abstract

Juvenile dermatomyositis (JDM) patients who test positive for the antimelanoma differentiation-associated gene 5 (MDA5) antibody have a poor prognosis because of rapidly progressing interstitial lung disease (ILD). However, agreement on the best treatment for this condition remains elusive. We encountered a 13-year-old girl with anti-MDA5 antibody-positive JDM who presented with arthritis and was already showing signs of ILD when she was admitted to the hospital. While cyclophosphamide (CY) is commonly used, it can cause gonadal disorders and other complications when administered to adolescent females. Consequently, we chose multitarget therapy, which includes tacrolimus and mycophenolate mofetil. Her ILD and skin symptoms gradually improved, and she was able to maintain remission and avoid CY administration for three years. We conducted a thorough literature review to determine the efficacy and safety of multitarget therapy for anti-MDA5 antibody-positive DM and JDM. Multitarget therapy shows promise as a potentially effective and relatively safe treatment. The ability to avoid CY, which is especially important for adolescent patients concerned about fertility preservation, highlights a significant benefit of this multitarget therapy for anti-MDA5 antibody-positive DM and JDM patients.

## Introduction

Juvenile dermatomyositis (JDM) is defined as pediatric DM that develops before the age of 16, and its clinical presentation may or may not be similar to adult DM [1]. Like adults with antimelanoma differentiation-associated gene 5 (anti-MDA5) antibody-positive DM, JDM patients with anti-MDA5 antibody positivity can develop ILD complications that can progress quickly and be fatal [1].

Anti-MDA5 antibody-positive DM is a rare idiopathic inflammatory myositis with classical DM-like cutaneous features but little or no involvement of the proximal muscle [1-3]. Anti-MDA5 antibody-positive DM is a difficult condition with a remarkably high mortality rate, owing primarily to the rapidly progressive interstitial lung disease (RP-ILD), which is most common in East Asia [3]. RP-ILD is defined as rapidly progressive dyspnea and hypoxemia with a worsening of radiologic interstitial lung changes within three months after the onset of respiratory symptoms [4]. The development of RP-ILD in anti-MDA5 antibody-positive JDM seems to be related to race, gender, and age, but no conclusions have been obtained [4]. Despite aggressive treatment, the six-month survival rate for RP-ILD patients with anti-MDA5 antibody positivity is less than 70% [2]. It is difficult to predict whether patients with anti-MDA5 antibody positivity will develop fatal disease progression at an early stage of the disease. 

Currently, there are no established guidelines for managing anti-MDA5 antibody-positive DM and JDM with ILD. Glucocorticoids, either alone or in combination with immunosuppressants, are regarded as the cornerstone of treatment [1,5]. Combination therapies with glucocorticoids and agents such as cyclophosphamide (CY), azathioprine, calcineurin inhibitors (CNIs) such as tacrolimus (Tac) and cyclosporin A, methotrexate, rituximab, and plasmapheresis are used, primarily based on uncontrolled retrospective analyses or case series. Treatment options are determined by various factors, including the patient’s age, gender, and ILD severity.

Among these treatment options, CY has been widely used and proven effective in the treatment of anti-MDA5 antibody-positive DM and JDM [6]. However, CY is associated with serious side effects such as hemorrhagic cystitis and bone marrow suppression. Long-term CY use has been associated with an increased risk of developing malignant tumors, particularly in adolescent patients, necessitating caution due to potential effects on fertility and reproductive function [7,8]. Given these concerns, alternative therapies such as immunomodulators and biologics are considered needed.

Therefore, multitarget therapy is a potential alternative to CY, hoping to avoid the associated side effects. In this context, we present the case of a 13-year-old anti-MDA5 antibody-positive JDM patient with ILD who was successfully treated with multitarget therapy, eliminating the need for CY administration. Additionally, we performed a retrospective literature review of DM and JDM patients with anti-MDA5 antibodies who received multitarget therapy.

## Case presentation

A 13-year-old girl came to our hospital with joint pain and a skin rash. She developed erythema on both fingers and right wrist pain two months ago, which did not go away despite topical steroids. She later reported bilateral finger pain, raised skin lesions, neck and lower back pain, and swelling in specific finger joints. The patient complained of occasional low-grade fever, decreased appetite, and joint stiffness, particularly in the morning, despite the absence of respiratory symptoms.

**Figure 1:**
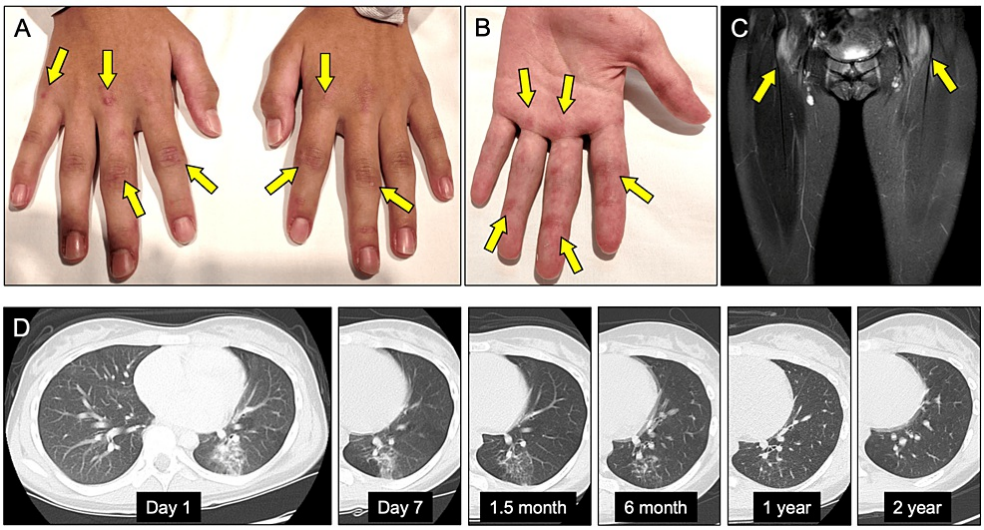
Clinical images in our case A. Gottron’s signs appear on the extensor side of finger joints. B. Reverse the Gottron sign on the palm side. C. Femoral MRI revealed symmetrical short-TI inversion recovery hyperintensity in the bilateral iliopsoas muscle. D. Results of chest computed tomography. At the time of diagnosis, a ground-glass shadow was seen in the left lower lobe. The ground-glass shadow in the left lower lobe gradually faded after treatment began and nearly vanished a year later. Over the next two years, there was no evidence of symptom recurrence or recurrence of ILD on imaging.

Her height was 154.7 cm, and she weighed 49.1 kg. The physical examination revealed distinct skin findings such as a malar rash, Gottron’s signs on finger joints (Figure 1A), reverse Gottron’s sign (Figure 1B), mechanic’s hands, Gottron’s papules on elbows and knees, and periungual erythema. No heliotrope rash was seen. Swelling and limited motion were observed in some peripheral joints. Manual muscle testing revealed full strength. Pulse oximetry showed 99% saturation in room air, and auscultation revealed clear lung sounds.

Laboratory tests (Table 1) revealed lymphocytopenia, elevated creatine kinase and aldolase levels, and a high ferritin level. Transaminase, Krebs von den Lungen-6 (KL-6), and matrix metalloprotease-3 levels were normal. The antinuclear antibody and the rheumatoid factor were negative. The anti-MDA5 antibody index was positive, whereas tests for anti-Mi-2, -ARS, -TIF1γ, and -Jo-1 were negative. Blood gas analysis revealed no hypoxemia or hypercapnia. Pulmonary function tests were normal. 

**Table 1:** Summary of quantitative laboratory investigations N.A.: Not analyzed

Parameters	Results						Reference and unit
	Initial presentation	Day 7	1.5 month	6 month	1 year	2 year	
White blood cells	3.41	9.46	9.79	5.91	5.85	6.06	3.3 - 8.8 x 10^3^/µL
Neutrophils	70.1	83.4	76.4	73.3	63.1	59.9	48.0 - 72.0 %
Lymphocytes	19.1	9.3	16.8	18.1	25.8	31.7	20.0 - 42.0 %
Hemoglobin	12.1	12.9	13.1	13.6	13.4	13.7	11.2 - 14.5 g/dL
Platelets	121	184	172	222	203	210	130 - 150 x 10^3^/µL
Erythrocyte sedimentation rate	22	12	4	2	2	2	<15 mm/hour
C-reactive protein (CRP)	0.07	0.02	<0.02	<0.02	<0.02	<0.02	<0.14 mg/dL
Ferritin	258	284	206	59	30	38	6.23 - 138 ng/mL
Total protein	7.5	7.2	7	6.7	7.1	7.6	6.7 - 8.3 g/dL
Albumin	4.3	3.8	3.9	4.6	4.6	4.9	4.0 - 5.0 g/dL
Blood urea nitrogen	9	21	19	13	12	15	8 - 22 mg/dL
Creatinine	0.42	0.7	0.38	0.45	0.6	0.65	0.50 - 0.80 mg/dL
Aspartate aminotransferase	43	18	17	12	16	19	13 - 33 IU/L
Alanine aminotransferase	32	31	26	11	15	17	6 - 27 IU/L
Lactate dehydrogenase	349	226	221	199	161	150	119 - 229 U/L
Creatine phosophokinase	171	20	14	39	55	41	30 - 135 IU/L (Female)
Aldorase	12	9	5	4	4	4	2 - 6 IU/L
Matrix metalloprotease-3	<16.0	N.A.	N.A.	N.A.	N.A.	N.A.	17.3 - 59.7 ng/mL
Immunoglobulin G (IgG)	1,557	N.A.	N.A.	614	1,050	N.A.	870 - 1,700 mg/dL
IgA	303	N.A.	N.A.	114	168	N.A.	110 - 410 mg/dL
IgM	106	N.A.	N.A.	47	90	N.A.	46 - 260 mg/dL
Krebs von den Lungen-6 (KL-6)	307	328	199	159	141	144	94.9 - 458.2 U/mL
Surfactant protein-D (SP-D)	85.7	N.A.	20.4	36.3	37.5	N.A.	<110 ng/mL
Anti-MDA5 antibody	605.0	325.0	53.0	23.0	28.0	45.0	<32.0 index
Anti-ARS antibody	6	N.A.	N.A.	N.A.	N.A.	N.A.	<25.0 index
Anti-TIF1γ antibody	<5	N.A.	N.A.	N.A.	N.A.	N.A.	<32.0 index
Anti-Mi-2 antibody	<5	N.A.	N.A.	N.A.	N.A.	N.A.	<53.0 index
Rheumatoid factor	<5	N.A.	N.A.	N.A.	N.A.	N.A.	<15 IU/mL
Antinuclear antibody test	<20	N.A.	N.A.	N.A.	N.A.	N.A.	<20 times
Varicella zoster virus IgG	34.2	N.A.	N.A.	N.A.	N.A.	N.A.	<2.0 (EIA)
Human cytomegalovirus IgG	3.3	N.A.	N.A.	N.A.	N.A.	N.A.	<2.0 (EIA)

Femoral MRI showed symmetrical short-TI inversion recovery hyperintensity in the bilateral iliopsoas, external obturator muscle, and gluteal muscles (Figure 1C). Chest computed tomography (CT) revealed interstitial pneumonia, which included a ground-glass shadow in the left lower lobe and increased density in the lower right lung (Figure 1D). Based on these results, the patient was diagnosed with anti-MDA5 antibody-positive JDM with ILD.

**Figure 2:**
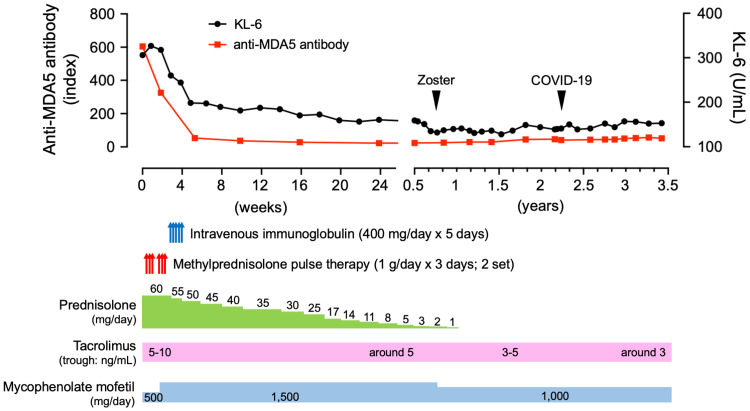
Changes in ILD after multitarget therapy Prednisolone at 60 mg/day, Tac at 4 mg/day, and MMF at 500 mg/day were started on the day of hospitalization. Furthermore, because the anti-MDA5 antibody was found to be positive, we immediately administered methylprednisolone pulse therapy (1 g/day x 3 days; 2 sets). Furthermore, intravenous immunoglobulin therapy was introduced. Following the completion of steroid pulse therapy, MMF was increased to 1,500 mg/day, and prednisolone was tapered at a rate of 5 mg every two weeks. Tac was adjusted to produce a trough concentration of 5 to 10 ng/mL. Levels of anti-MDA5 antibody and KL-6 gradually decreased and had nearly returned to normal by 1.5 months. Subsequently, at 10 months, the patient developed herpes zoster, necessitating the discontinuation of oral steroids and a decrease in the dosage of MMF. The blood concentration of Tac was also adjusted to be between 3 and 5 ng/mL. Despite contracting COVID-19 2.5 years later, anti-MDA5 antibody levels remained unchanged, and ILD did not worsen. Currently, after 3.5 years, the condition has stabilized. Image Credits: This figure was originally created by T. Yokoyama.

Treatment began on the first day of admission (day 1) with the administration of prednisolone at 60 mg/day, Tac (3 mg/day), and MMF (500 mg/day) (Figure 2A). The first course of methylprednisolone pulse therapy (1 g/day for three days) began on day four. On day seven, the improvement in shadows on chest CT was confirmed (Figure 1D). Then, a second course of methylprednisolone pulse therapy was started, with MMF increased to 1,500 mg/day. Furthermore, intravenous immunoglobulin (400 mg/day for five days) was administered beginning on day 15. KL-6 levels, after peaking on day seven, fell steadily (Table 1). During the treatment period, neither respiratory symptoms nor chest CT findings worsened. There were no side effects after completing intravenous immunoglobulin treatment and two courses of methylprednisolone pulse therapy. Prednisolone was gradually tapered on day 29, and she was discharged on day 39 after confirmation of stable blood test results, respiratory function, and no exacerbations of symptoms.

Since then, the patient has been gradually tapering off prednisolone (5 mg per two weeks) as an outpatient, with no worsening of symptoms, laboratory findings, or imaging results. Tac was set to a trough level of 5 to 10 ng/mL at the start of treatment. After six months, the dose was reduced to approximately 5 ng/mL. The anti-MDA5 antibody titer gradually decreased and became negative about four months after starting treatment (Table 1, Figure 2). At 10 months, the patient developed herpes zoster and was treated with valaciclovir, which reduced the MMF dosage to 1,000 mg/day. Tac’s blood concentration was also adjusted to a trough level of 3 to 5 ng/mL. After one year, prednisolone was completely discontinued. Shadows disappeared on a chest CT, and they did not reappear two years later on another chest CT.

Currently, the patient has been receiving Tac (2 mg/day; trough around 3 ng/mL) and MMF (1,000 mg/day) for a total of three years. At 2.5 years after beginning treatment, the patient contracted COVID-19, resulting in a high fever and severe fatigue that required a one-week hospital stay. Importantly, ILD did not worsen during this period. The patient has maintained a good condition with no recurrences, and CY has not yet been used.

## Discussion

We present the case of a 13-year-old patient with anti-MDA5 antibody-positive JDM and ILD who achieved good disease control without CY using multitarget therapy. JDM treatment options vary depending on severity, ranging from mild cases treated with steroid ointment alone to more severe cases requiring steroid pulse therapy in combination with intravenous CY pulse therapy. When deciding on a treatment strategy, it is critical to consider the presence of interstitial lesions detected on a chest CT. In this patient’s case, a chest CT revealed an interstitial lesion, and anti-MDA5 antibody- positivity was linked to a high risk of RP-ILD, necessitating intensive treatment.

Typically, in the presence of ILD, treatment consists of methylprednisolone pulse therapy combined with CNIs or CY, or a combination of three drugs [6]. However, the use of CY can be difficult for adolescent girls due to concerns about potential side effects such as fertility and carcinogenicity [7,8].

In our case, the patient was in adolescence, and she and her parents desired to avoid CY. According to the imaging, her ILD was relatively mild. Kobayashi et al. demonstrated that the risks for RP-ILD include the presence of MDA5 antibodies, ferritin levels, KL-6 levels, and interleukin-18 concentration (which was not measured in our case) [9]. Among these, the presence of MDA5 antibodies and ferritin levels indicated a similar level to cases that progress to RP-ILD, making it difficult to determine at the initial diagnosis whether the patient would progress to RP-ILD in the future. Therefore, we began treatment with methylprednisolone pulse therapy and multitarget therapy using MMF and Tac before administering CY, closely monitoring respiratory symptoms thereafter. Although plans were made to administer CY if respiratory lesions worsened, this was not necessary, and the patient achieved remission.

Tac, a CNI, can inhibit lymphoid gene transcription and have potent immunosuppressive effects by targeting both cellular and humoral immune mechanisms [10,11]. Multitarget therapy can effectively address multiple disease pathogenic factors, overcoming the limitations of single-target medications [10,11]. It can also modulate the network system of multiple interconnected factors, resulting in synergistic effects and optimal treatment outcomes while preventing drug resistance [10,11].

Mycophenolate mofetil (MMF), an inosine-5'-monophosphate dehydrogenase (IMPDH) inhibitor, selectively inhibits T and B lymphocyte proliferation, suppresses antibody synthesis, and prevents the formation of endothelial adhesion factors [12]. It also inhibits the proliferation of arterial smooth muscle and endothelial cells, making it useful for treating vascular inflammatory lesions [12]. Recent evidence has shown the efficacy of MMF as a steroid-free treatment for ILD associated with polymyositis and DM in a few case series [13]. MMF is recommended by the Childhood Arthritis and Rheumatology Research Alliance and the Single Hub and Access Point for Pediatric Rheumatology in Europe (SHARE) for JDM cases that do not respond to combination therapy with glucocorticoids and methotrexate [5,14]. Furthermore, MMF is considered equally effective as CY in treating lupus nephritis, with the added benefit of preserving fertility, in guidelines such as Kidney Disease Improving Global Outcomes (KDIGO) 2021 and European Alliance of Associations for Rheumatology (EULAR) 2019 for systemic lupus erythematosus (SLE)/lupus nephritis [15,16].

Bao et al. first described the efficacy of combination therapy using Tac and MMF for lupus nephritis class V+IV, coining the term “multitarget therapy” [17]. Subsequently, widespread evidence of the efficacy of multitarget therapy, which combines CNIs (Tac or CyA) and MMF, has been observed in the treatment of SLE and lupus nephritis.

**Figure 3:**
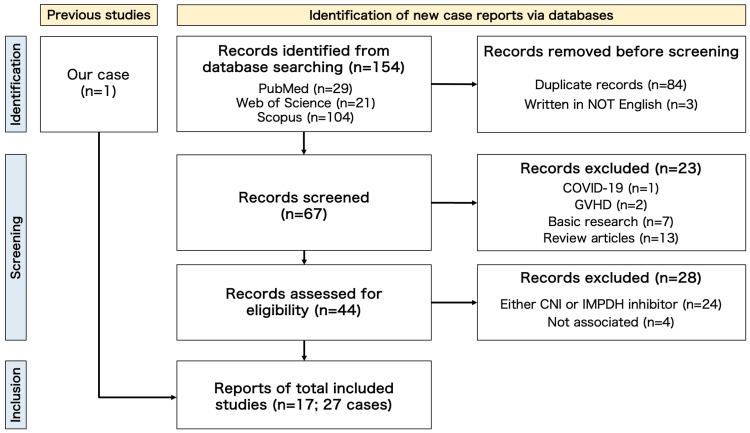
Flow diagram (PRISMA) of a literature review for multitarget therapy in patients with anti-MDA5 antibody-positive DM and JDM Our initial keyword-based search yielded 154 papers. Out of these, 84 duplicate papers were removed, and three papers were excluded because they were not written in English. After reviewing the abstracts, we excluded one paper describing COVID-19 and two papers describing graft-versus-host diseases. We then proceeded to evaluate the remaining 64 full-text papers obtained from various sources, including the Web, Kanazawa University Library, and the National Diet Library. Among these 64 papers, seven were focused on basic research, while 13 were review articles, which were excluded from our analysis. Furthermore, 24 papers dealt solely with either calcineurin inhibitors (CNI) or inosine monophosphate dehydrogenase (IMPDH) inhibitors, which were irrelevant to our study. Four papers were determined to be unrelated to DM and JDM. Consequently, we identified 16 papers that described a total of 28 cases. Among these cases, two were excluded because they involved switching from CNIs to IMPDH inhibitors rather than combining both drugs. Finally, we included 16 papers and our case report, for a total of 27 cases in our analysis.

To evaluate the effectiveness and safety of multitarget therapy in patients with anti-MDA5 antibody-positive DM and JDM without the use of CY, we conducted a comprehensive literature review (Figure 3; PRISMA diagram).

We conducted a systematic search of MEDLINE/PubMed, Web of Science, and Scopus using specific keywords. These keywords were divided into three categories: a) “dermatomyositis”, b) “MDA5” or “melanoma differentiation-associated gene 5” and c) “MMF” or “mycophenolate mofetil” or “mizoribine” or “multitarget therapy”. We used one keyword from each category in our literature search. All of the retrieved literature was considered for inclusion. Duplicate articles, non-English articles, and articles unrelated to the topic of our study were excluded at the abstract level. Ultimately, we found 16 papers and 26 cases that met the inclusion criteria. Table 2 presents a summary of the remaining 27 cases, including our own.

**Table 2:** Results of a literature review about multitarget therapy for anti-MDA5 antibody-positive DM and JDM F: female, M: male, +: positive, N.D.: not described, ILD: interstitial lung disease, PTX: pheumothorax, RP-ILD: rapidly progressive-ILD, CNI: calcineurin inhibitor, CyA: cyclosporine A, Tac: tacrolimus, In: Initial, Add: Additional medication, IMPDH: inosine 5’-monophosphate dehydrogenase, MMF: mycophenolate mofetil, MZR: mizoribine, CY: cyclophosphamide, IVIG: intravenous high-dose immunoglobulin therapy, RTX: rituximab, MTX: methotrexate, AZA: azathioprine, HCQ: hydroxychlorquine, ●: used, -: not used

Author(s)/year	Age	Sex	Race	anti-MDA5 Ab (index)	ferritin (ng/mL)	KL-6 (U/mL)	CT findings and progress	CNI	IMPDH inhibitor	Steroid pulse	Steroids	CY	IVIG	RTX	Others	Outcome	Adverse effect
Multitarget therapy WITHOUT CY																	
*Huang K, et al*. 2019 [18].	21	F	Indian	+	N.D.	N.D.	Chronic-ILD	Tac(In)	MMF(In)	-	●	-	-	●	AZA	Alive	N.D.
	27	F	Chinese	+	N.D.	N.D.	Chronic-ILD and PTX	Tac(In)	MMF(In)	-	●	-	●	●	AZA	Alive	N.D.
	59	F	Filipino	+	N.D.	N.D.	Chronic-ILD and PTX	CyA(In)	MMF(In)	-	●	-	-	●	AZA + HCQ	Alive	N.D.
*So H, et al*. 2018 [19].	38	M	Chinese	+	N.D.	N.D.	RP-ILD	Tac(In)	MMF(In)	-	●	-	●	●	-	Alive	Chest infection
*Hoa S, et al*. 2018 [20].	50	M	White	+	849	N.D.	RP-ILD	Tac(Add)	MMF(In)	-	●	-	-	-	HCQ	Alive	N.D.
	55	F	Asian	+	1630	N.D.	RP-ILD	Tac(In)	MMF(Add)	●	●	-	●	●	HCQ	Alive	N.D.
	61	F	White	+	370	N.D.	RP-ILD	Tac(In)	MMF(In)	●	●	-	-	-	HCQ	Alive	N.D.
*Suda M, et al*. 2017 [21].	63	F	Japanese	>150	306	1276	Early stage	Tac(In)	MZR(In)	-	●	-	-	-	-	Alive	No serious adverse effect
	45	M	Japanese	>150	366	223	Early stage	Tac(In)	MZR(In)	-	●	-	-	-	-	Alive	No serious adverse effect
*Intapiboon P, et al*. 2020 [22].	36	F	Thai	+	1102	N.D.	RP-ILD	CyA	MMF	●	●	-	-	●	-	Died	N.D.
*Takada T, et al*. 2015 [23].	29	F	Japanese	91.1	N.D.	N.D.	N.D.	CyA	MMF	●	●	-	-	-	-	Alive	N.D.
	72	M	Japanese	182.4	N.D.	N.D.	RP-ILD	CyA	MMF	●	●	-	-	-	-	Died	N.D.
Our case	13	F	Japanese	605	258	307	Early stage	Tac(In)	MMF(In)	●	●	-	●	-	-	Alive	Zoster
Multitarget therapy WITH CY																	
*Muramatsu T, et al*. 2021 [24].	39	F	Japanese	157	22	699	RP-ILD	CyA(In) → Tac	MMF(Add)	-	●	●	-	-	-	Alive	N.D.
*Yeung TW, et al*. 2021 [25].	16	F	Chinese	+	893	N.D.	RP-ILD	Tac(In)	MMF(Add)	●	●	●	-	●	MTX	Alive	Elevated intraocular pressure
*Saito T, et al*. 2021 [26].	46	M	Japanese	>150	1388	801	RP-ILD	Tac(In) → CyA	MMF(Add)	●	●	●	-	●	Apheresis	Alive	PRES
*Ge Y, et al*. 2021 [27].	36	F	Asian	+	77	N.D.	RP-ILD	CNI	MMF(Add)	-	●	●	-	●	-	Alive	N.D.
*So H, et al*. 2018 [19].	49	F	Chinese	+	N.D.	N.D.	RP-ILD	CyA(In)	MMF(In)	-	●	●	●	●	-	Alive	Vasculitic ulcer wound infection
	50	M	Chinese	+	N.D.	N.D.	RP-ILD	Tac(In)	MMF(In)	-	●	●	-	●	-	Alive	Chest infection
*Clottu A, et al*. 2012 [28].	68	F	European	+	805	N.D.	RP-ILD	CyA(Add)	MMF(Add)	-	●	●	●	●	HCQ	Alive	N.D.
*Hayashi M, et al*. 2017 [29].	59	M	Japanese	382	N.D.	N.D.	RP-ILD	CyA(In)	MMF(Add)	●	●	●	-	-	-	Alive	CMV antigenemia, pneumomediastinum, and renal dysfunction
*Tokunaga K, et al*. 2017 [30].	71	F	Japanese	+	N.D.	N.D.	RP-ILD	Tac(In) → CyA	MMF(Add)	●	●	●	-	●	-	Died	N.D.
*Intapiboon P, et al*. 2020 [22].	59	F	Thai	+	2129	N.D.	RP-ILD	CyA	MMF	●	●	●	-	-	-	Alive	N.D.
*Chino H, et al*. 2019 [31].	64	F	Japanese	37.3	N.D.	1472	RP-ILD	CNI	MMF	●	●	●	●	-	Apheresis	Died	N.D.
	63	F	Japanese	567.9	567.9	624	RP-ILD	Tac	MMF	●	●	●	●	●	-	Alive	N.D.
*Sulaiman W, et al*. 2021 [32].	44	F	Indian	+	1599	N.D.	RP-ILD	Tac(In)	MMF(In)	●	●	●	-	-	-	Alive	N.D.
*Hisanaga J, et al*. 2017 [33].	57	F	Japanese	+	697	869	RP-ILD	CyA(In)	MMF(Add)	-	●	●	●	●	Apheresis	Alive	N.D.

Among the 27 cases, ours was the youngest. Among these cases, 13 avoided using CY, while the remaining 14 used it alongside CNI and IMPDH inhibitors. Of the 13 cases avoided by CY, 11 had a favorable outcome. Furthermore, rituximab was used in 15 cases, with 5 of the 13 cases that did not require CY opting for it.

It is unclear whether multitarget therapy is superior to combination therapy with CY due to the lack of a controlled comparison in this study. However, it is worth noting that multitarget therapy alone is not contraindicated, as some cases have combined it with CY or rituximab. One concern with multitarget therapy is the risk of excessive immunosuppression, which can lead to opportunistic infections. Many papers did not mention adverse effects, but those that did frequently reported infections. Our case also developed herpes zoster during the 2.5 years of multitarget therapy. This occurrence could be a side effect of immunosuppression, emphasizing the importance of constantly monitoring for infections while undergoing this multitarget therapy.

## Conclusions

In conclusion, multitarget therapy for anti-MDA5 antibody-positive DM and JDM is a potentially effective treatment that can be administered with relative safety. Particularly, the ability to avoid CY is a significant advantage of multitarget therapy, especially for adolescent patients who are concerned about fertility preservation. However, it was not elucidated whether multitarget therapy is effective against not only ILD but also RP-ILD and severe complications without using CY. In the future, large clinical trials comparing multitarget therapy and CY should be conducted, and the establishment of more effective and safer treatments for anti-MDA5 antibody-positive DM and JDM patients is desired.
